# Uncovering Language Disparity of ChatGPT on Retinal Vascular Disease Classification: Cross-Sectional Study

**DOI:** 10.2196/51926

**Published:** 2024-01-22

**Authors:** Xiaocong Liu, Jiageng Wu, An Shao, Wenyue Shen, Panpan Ye, Yao Wang, Juan Ye, Kai Jin, Jie Yang

**Affiliations:** 1 Eye Center The Second Affiliated Hospital Zhejiang University Zhejiang China; 2 School of Public Health Zhejiang University School of Medicine Zhejiang China

**Keywords:** large language models, ChatGPT, clinical decision support, retinal vascular disease, artificial intelligence

## Abstract

**Background:**

Benefiting from rich knowledge and the exceptional ability to understand text, large language models like ChatGPT have shown great potential in English clinical environments. However, the performance of ChatGPT in non-English clinical settings, as well as its reasoning, have not been explored in depth.

**Objective:**

This study aimed to evaluate ChatGPT’s diagnostic performance and inference abilities for retinal vascular diseases in a non-English clinical environment.

**Methods:**

In this cross-sectional study, we collected 1226 fundus fluorescein angiography reports and corresponding diagnoses written in Chinese and tested ChatGPT with 4 prompting strategies (direct diagnosis or diagnosis with a step-by-step reasoning process and in Chinese or English).

**Results:**

Compared with ChatGPT using Chinese prompts for direct diagnosis that achieved an *F*_1_-score of 70.47%, ChatGPT using English prompts for direct diagnosis achieved the best diagnostic performance (80.05%), which was inferior to ophthalmologists (89.35%) but close to ophthalmologist interns (82.69%). As for its inference abilities, although ChatGPT can derive a reasoning process with a low error rate (0.4 per report) for both Chinese and English prompts, ophthalmologists identified that the latter brought more reasoning steps with less incompleteness (44.31%), misinformation (1.96%), and hallucinations (0.59%) (all *P*<.001). Also, analysis of the robustness of ChatGPT with different language prompts indicated significant differences in the recall (*P*=.03) and *F*_1_-score (*P*=.04) between Chinese and English prompts. In short, when prompted in English, ChatGPT exhibited enhanced diagnostic and inference capabilities for retinal vascular disease classification based on Chinese fundus fluorescein angiography reports.

**Conclusions:**

ChatGPT can serve as a helpful medical assistant to provide diagnosis in non-English clinical environments, but there are still performance gaps, language disparities, and errors compared to professionals, which demonstrate the potential limitations and the need to continually explore more robust large language models in ophthalmology practice.

## Introduction

The global population of individuals with visual impairments exceeded 2.2 billion in 2019 and continues to rise [1]. As the leading causes of blindness, retinal vascular diseases are characterized by a complex array of clinical manifestations [2]. Fundus fluorescein angiography (FFA), which uses an injected fluorescent dye to examine circulation in the retina and choroid, is a specialized ophthalmic test used to visualize the retinal vasculature [3]. In practice, interpreting FFA results and making a diagnosis requires laborious analysis by experienced ophthalmologists.

In recent years, significant developments in deep learning approaches, which are extensively utilized, have rendered them a promising way for auxiliary diagnosis of retinal vascular diseases. The existing research has mainly focused on developing convolutional neural network algorithms for lesion detection in FFA images [4-8], such as microaneurysms, leakages, nonperfusion areas, and neovascularization. Further, some studies focused on automatically generating FFA reports [9,10], which can highlight abnormalities for ophthalmologists and provide a theoretical basis for disease diagnosis. However, few studies were devoted to the diagnosis of retinal vascular disease based on FFA reports. The main challenges of using natural language processing to diagnose retinal vascular diseases can be summarized as follows: (1) different interpretation of FFA images by different ophthalmologists, (2) varied ophthalmological terms contained in FFA reports, (3) time-varying imaging features contained in FFA reports, and (4) smaller data volume caused by the high cost and possible side effects of FFA.

Recently, large language models (LLMs) like ChatGPT [11] have demonstrated exceptional performance in various tasks due to their rich internal knowledge and strong deductive reasoning abilities [12-16]. However, the related research within the medical field primarily focuses on knowledge assessment [17-20], and a comprehensive evaluation of ChatGPT’s capabilities in ophthalmology for disease diagnosis is lacking. Additionally, although existing LLMs demonstrate impressive cross-language understanding abilities, they may lead to significant disparities in non–English-specific fields because they were primarily trained on English corpora [21,22]. Therefore, in this study, by exploring ChatGPT’s ability to understand Chinese FFA reports, our objectives were to evaluate ChatGPT’s diagnostic performance and inference abilities for retinal vascular diseases in a non-English clinical environment and to find appropriate prompt strategies under these scenarios.

## Methods

### Data Preparation

We collected 1226 Chinese FFA reports and the corresponding clinical diagnoses of 728 patients from the Eye Center of the Second Affiliated Hospital of Zhejiang University (SAHZU) between August 2016 and September 2021. The clinical diagnosis of each eye was either classified as normal or one of the 6 primary retinal vascular diseases: diabetic retinopathy (DR), wet age-related macular degeneration, central serous chorioretinopathy (CSC), branch retinal vein occlusion (BRVO), central retinal vein occlusion (CRVO), and Vogt-Koyanagi-Harada disease (VKH). The clinical diagnosis was based on clinical information from the patients, primarily the FFA images and reports.

### Ethical Considerations

Ethical approval was obtained from the Ethics Committee in the SAHZU School of Medicine (2019-428). This research involves medical records data. We ensured that the medical records were deidentified and all private information was removed. The Institutional Review Board agreed to share access to the data with third parties, including sending it through application programing interfaces (APIs) provided by companies like OpenAI, or using it on web-based platforms like ChatGPT.

### Diagnosis of Retinal Vascular Diseases Using ChatGPT

To diagnose the patient’s eye status based on the FFA report with ChatGPT, we designed a fixed instruction that concatenates the patient’s FFA report as the whole prompt for ChatGPT. The instruction consists of a specific task description and all alternative conditions. To fully exploit the potential of ChatGPT, we implemented different prompting strategies to investigate the potential effect and find the most appropriate way to apply it. First, we used the direct prompting strategy that requires ChatGPT to directly output the final option without explanations. Second, inspired by chain-of-thought prompting technology [23], we adopted a step prompting strategy to elicit the detailed reasoning process, which provides interpretability for the disease diagnosis. Finally, ChatGPT was primarily trained on English corpora and may have difficulty recognizing instructions and FFA reports in Chinese, as well as making use of internal knowledge. Therefore, we also rewrote the prompts in English while keeping the FFA reports in Chinese to conduct code-switching prompt examination. Therefore, we mainly investigated 4 prompt strategies: Direct-Chinese, Step-Chinese, Direct-English, and Step-English. The detailed prompts can be found in Multimedia Appendix 1.

**Figure 1:**
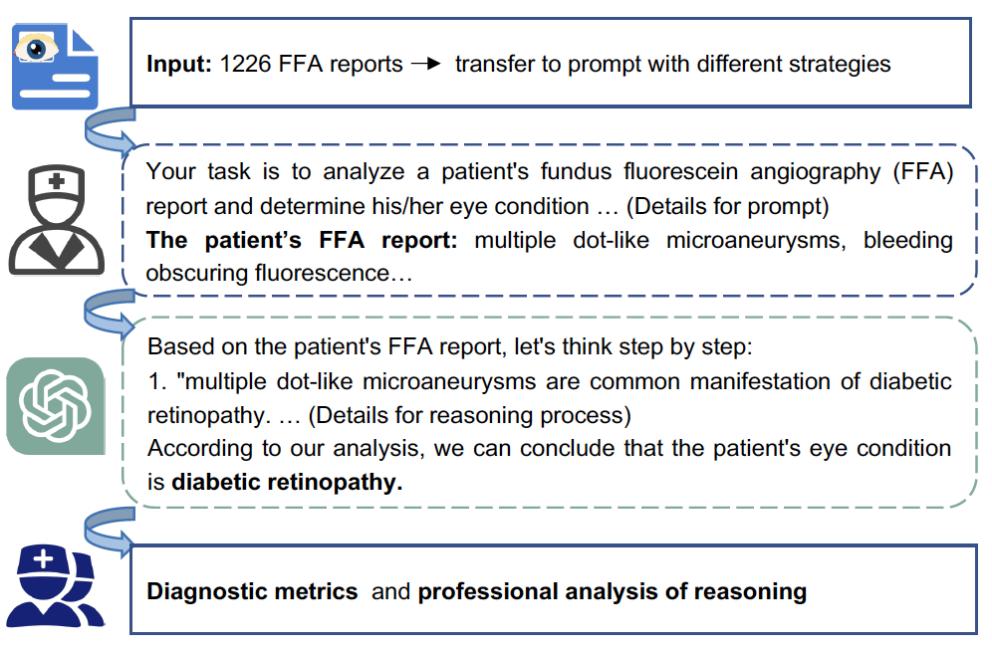
The overall workflow.

To avoid the randomness of ChatGPT’s response, we set the inference temperature to 0 so as to choose the greedy decoding strategy via the API, making the response more focused and deterministic. Furthermore, we evaluated the robustness of ChatGPT to different languages by calculating the average performance of ChatGPT using different prompting methods. All tests were conducted on the same version of GPT3.5-Turbo-0301 using the official API of OpenAI. Figure 1 shows the overall workflow.

### Measurements and Definitions

We designed a systematic evaluation scheme to assess the performance of ChatGPT. In addition to diagnostic performance, we also incorporated a combination of inference ability, omission of information, hallucinations, misinformation, and inconsistency assessments to evaluate the ChatGPT’s reasoning process, as follows:

1. Diagnostic performance: precision, recall, and *F*_1_-score.
2. Inference ability [24-26]: the total reasoning steps, the number of reasoning errors, and the incompleteness of the inference process.
3. Omission of information [27]: whether crucial information from the original report was overlooked.
4. Hallucinations [25,28]: whether ChatGPT generated medical findings that were not present in the original report.
5. Misinformation [29,30]: whether the output of ChatGPT quoted incorrect prior knowledge.
6. Inconsistency [30]: whether the reasoning result was inconsistent with the reasoning process.

For diagnostic evaluation, precision, recall, and *F*_1_-score were calculated based on ChatGPT’s responses and gold clinical diagnosis. Additionally, to evaluate the diagnostic performance of ChatGPT, 2 ophthalmologists and 2 ophthalmology interns with 2 years of clinical experience from SAHZU were invited to diagnose 100 FFA reports, which were randomly selected according to the proportion of diseases. In terms of the evaluation on ChatGPT’s inference ability, the last 5 measurements were evaluated on the responses to the Step-Chinese and Step-English prompts by 2 ophthalmologists from SAHZU. We randomly selected 509 FFA reports (no more than 100 for each disease) and the corresponding ChatGPT outputs for evaluation. Before the formal evaluation, the ophthalmologists were requested to conduct an annotation with training and achieved a final interannotator agreement up to 94%. The statistical analysis between the Chinese and English prompts was performed with the use of Chi-square tests for categorical measurements and Student *t* tests or Wilcoxon rank-sum tests for continuous measurements, as appropriate. A 2-sided *P*<.05 was considered statistically significant.

## Results

### Characteristics of ChatGPT’s Responses

The characteristics of the FFA reports and responses by ChatGPT are listed in Table 1. Direct-Chinese and Direct-English prompts received responses that directly provided the final options, and their mean (SD) lengths were 19.2 (4.4) tokens and 5.7 (1.7) tokens, respectively, while Step-Chinese and Step-English prompts received responses that provided the detailed reasoning process, and their mean (SD) lengths were 118.4 (71.8) tokens and 100.5 (36.9) tokens, respectively. Examples of different prompts and their responses are presented in Multimedia Appendix 1.

**Table 1:** Characteristics of the FFA reports and ChatGPT’s responses (N=1226).

Category	Count, n	Report length (tokens), mean (SD)	Response length (tokens), mean (SD)			
			Direct-Chinese	Direct-English	Step-Chinese	Step-English
Normal	117	10.5 (2.4)	14.2 (2.0)	5.4 (1.6)	86.6 (52.7)	64.6 (23.7)
DR^a^	717	46.4 (12.1)	19.5 (5.0)	5.7 (1.6)	124.0 (81.2)	100.9 (32.1)
wetAMD^b^	183	31.1 (11.2)	20.5 (1.5)	6.1 (1.6)	108.9 (46.4)	114.4 (44.7)
CSC^c^	73	29.9 (6.7)	19.3 (2.7)	6.3 (1.9)	146.7 (78.7)	127.4 (41.5)
BRVO^d^	63	44.7 (11.1)	19.8 (2.0)	5.2 (1.8)	106.5 (23.9)	87.2 (24.1)
CRVO^e^	38	50.6 (10.5)	20.7 (3.2)	4.8 (1.8)	134.5 (52.1)	91.4 (22.6)
VKH^f^	35	34.7 (13.5)	19.9 (2.3)	5.3 (1.4)	105.4 (43.9)	116.5 (41.4)
Overall	1226	39.4 (15.9)	19.2 (4.4)	5.7 (1.7)	118.4 (71.8)	100.5 (36.9)

^a^DR: diabetic retinopathy.

^b^wetAMD: wet age-related macular degeneration.

^c^CSC: central serous chorioretinopathy.

^d^BRVO: branch retinal vein occlusion.

^e^CRVO: central retinal vein occlusion.

^f^VKH: Vogt-Koyanagi-Harada disease.

### Diagnostic Performance

The Direct-English prompts achieved an overall precision of 79.61%, recall of 83.12%, and *F*_1_-score of 80.05%, which was 9.58% higher than that achieved by the Direct-Chinese prompts (Table 2). The diagnostic performance varied significantly for each disease category. ChatGPT performed better in the normal and DR categories, with the *F*_1_-scores exceeding 80%, but performed worse in the VKH and CSC categories, achieving *F*_1_-scores of less than 4%. Additionally, the *F*_1_-score in the BRVO category varied greatly, from 54.35% for Direct-Chinese prompts to 74.51% for Direct-English prompts.

**Table 2:** Diagnostic performance of ChatGPT across various disease categories on the FFA reports.

Category	Direct-Chinese (%)			Direct-English (%)				Step-Chinese (%)				Step-English (%)		
	P^a^	R^b^	*F*_1_	P	R	*F*_1_	P		R	*F*_1_	P		R	*F*_1_
Normal	100	85.47	92.17	100	88.03	93.64	98.39		52.14	68.16	97.37		94.87	96.1
DR^c^	91.55	72.52	80.93	91.05	95.12	93.04	85.07		95.4	89.94	82.13		93.58	87.48
wetAMD^d^	44.72	87.98	59.3	59.92	80.87	68.84	63.58		60.11	61.8	60		34.42	43.75
CSC^e^	4.35	2.74	3.36	33.33	1.37	2.63	34.15		19.18	24.56	50		6.85	12.05
BRVO^f^	41.32	79.37	54.35	63.33	90.47	74.51	83.61		80.95	82.26	67.95		84.13	75.18
CRVO^g^	93.1	71.05	80.6	84.85	73.68	78.87	41.27		68.42	51.49	58.33		73.68	65.12
VKH^h^	0	0	0	0	0	0	0		0	0	0		0	0
Overall	75.03	70.15	70.47	79.61	83.12	80.05	76.24		77.16	75.61	74.56		75.94	73.46

^a^P: precision.

^b^R: recall.

^c^DR: diabetic retinopathy.

^d^wetAMD: wet age-related macular degeneration.

^e^CSC: central serous chorioretinopathy.

^f^BRVO: branch retinal vein occlusion.

^g^CRVO: central retinal vein occlusion.

^h^VKH: Vogt-Koyanagi-Harada disease.

**Figure 2:**
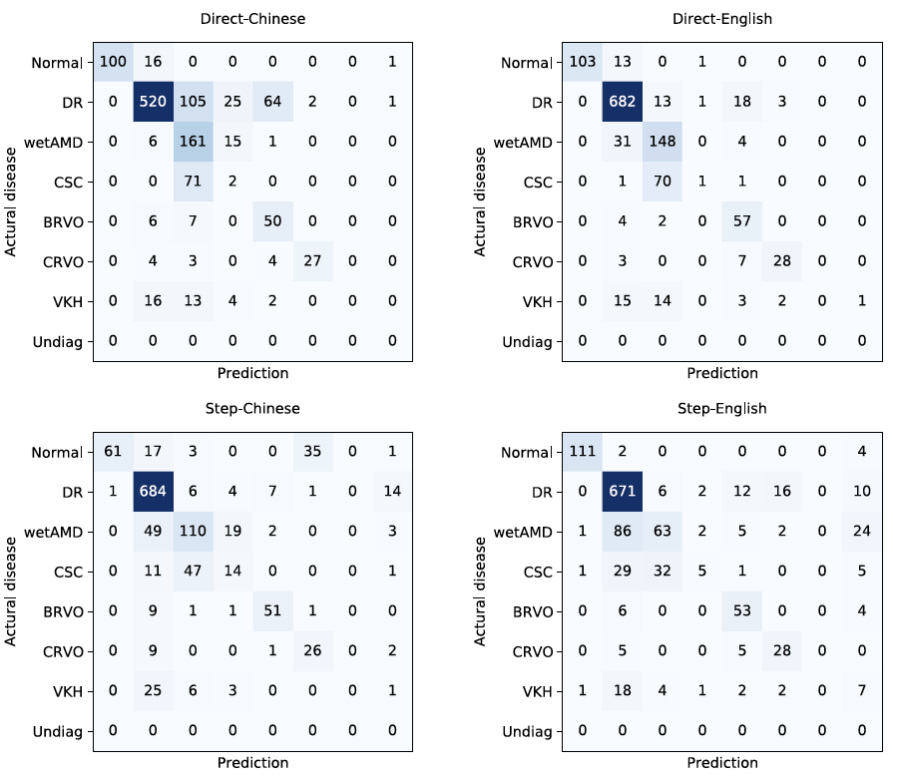
Confusion matrices of ChatGPT for the 4 prompting strategies. BRVO: branch retinal vein occlusion; CSC: central serous chorioretinopathy; CRVO: central retinal vein occlusion; DR: diabetic retinopathy; Undiag: undiagnosed; VKH: Vogt-Koyanagi-Harada disease; wetAMD: wet age-related macular degeneration.

In contrast, the Step-Chinese prompts achieved an overall precision of 76.24%, recall of 77.16%, and *F*_1_-score of 75.61%, which was 2.15% higher than that achieved by ChatGPT for Step-English prompts. Compared with Direct-Chinese prompts, the *F*_1_-score for Step-Chinese prompts was increased by 5.14% and provided the reasoning process, which is crucial for disease diagnosis. However, the diagnostic performance of Step-Chinese prompts in the normal and CRVO categories was far worse than that of Direct-Chinese prompts. This is mainly because, with Step-Chinese prompts, ChatGPT generated hallucinations for FFA reports in the normal category, which were wrongly diagnosed as CRVO. Figure 2 further demonstrates the confusion matrices of ChatGPT for the 4 prompting strategies.

**Figure 3:**
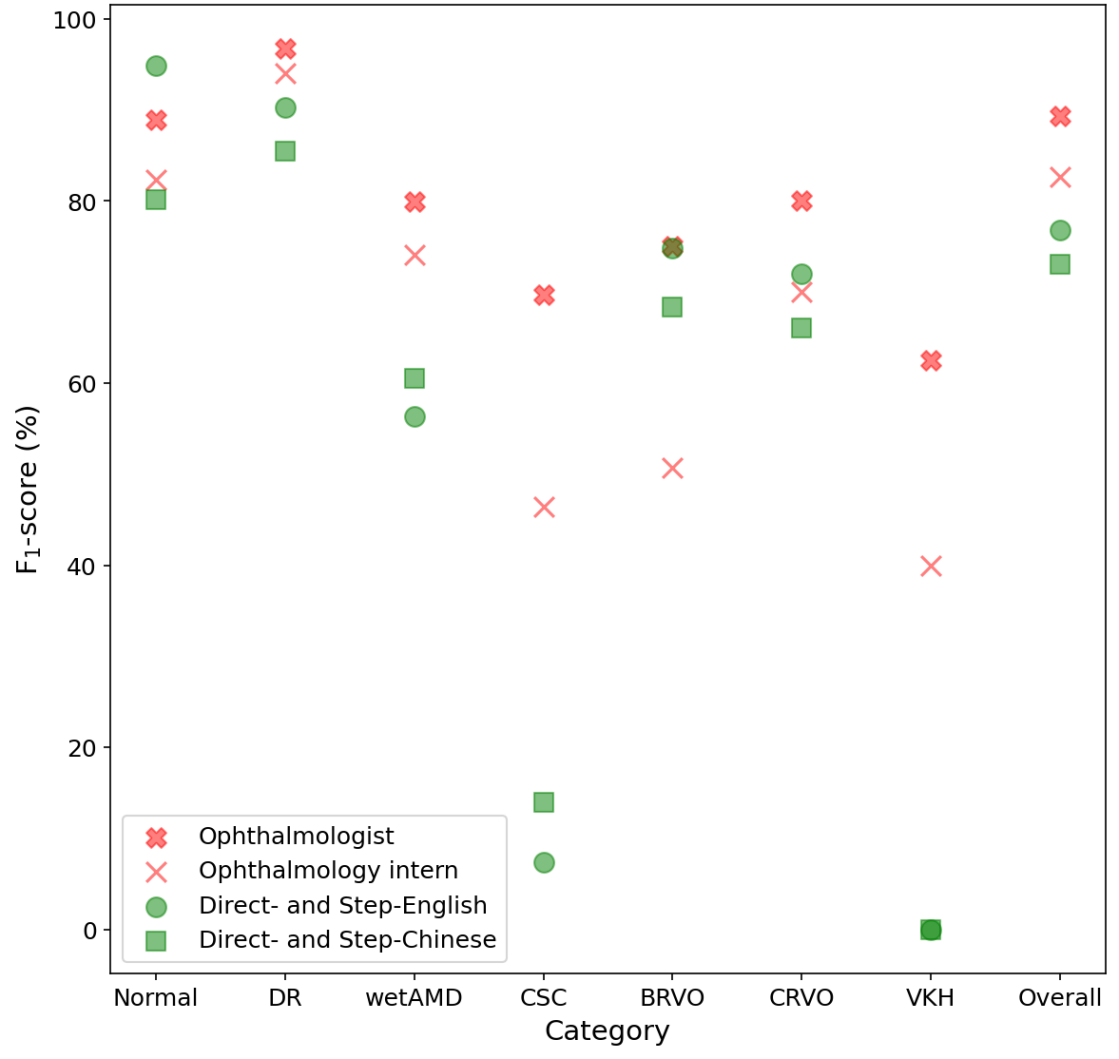
Diagnostic performance of humans and ChatGPT. BRVO: branch retinal vein occlusion; CSC: central serous chorioretinopathy; CRVO: central retinal vein occlusion; DR: diabetic retinopathy; VKH: Vogt-Koyanagi-Harada disease; wetAMD: wet age-related macular degeneration.

Figure 3 shows the average *F*_1_-score of ophthalmologists, ophthalmology interns, ChatGPT with English prompts (Direct-English and Step-English), and ChatGPT with Chinese prompts (Direct-Chinese and Step-Chinese). Although ChatGPT performed better than experts for some disease types (eg, Direct-English and Step-English prompts for the normal and CRVO categories and all prompts for the BRVO category), the overall performance of ophthalmologists was the best (89.35%), followed by ophthalmology interns (82.69%), ChatGPT with Direct-English and Step-English prompts (76.76%), and ChatGPT with Direct-Chinese and Step-Chinese prompts (73.04%).

### Inference Ability

Table 3 presents the outcomes of ChatGPT’s inference ability, as evaluated by 2 ophthalmologists. The results of continuous measurements were presented descriptively as mean (SD) values. Based on the 509 FFA reports, Step-English prompts exhibited a tendency to require more reasoning steps for each report (*P*<.001, Wilcoxon rank-sum test). Although the average number of inference errors was similar (0.4 per report) between Step-Chinese and Step-English prompts (*P*=.88, Wilcoxon rank-sum test), Step-English prompts brought less incompleteness (44.31%), hallucinations (0.59%), and misinformation (1.96%) (all *P*<.001, Chi-square test). Instead, Step-Chinese prompts, which involved fewer reasoning steps, were more prone to having key information from the original report overlooked during the reasoning process, although this difference lacked statistical significance (*P*=.68, Chi-square test). In addition, a few generated diagnoses were marked as inconsistent with the reasoning process, with no statistical difference observed between Step-Chinese and Step-English prompts. Overall, compared with Step-Chinese prompts, ChatGPT demonstrated stronger inference abilities for Step-English prompts, particularly with regard to the average number of reasoning steps, incompleteness, hallucinations, and misinformation.

**Table 3:** Inference ability of ChatGPT in the diagnosis of retinal vascular diseases.

Measurement	Step-Chinese	Step-English	*P* value^a^
Reasoning steps per report, mean (SD)	1.4 (0.8)	2.6 (1.5)	<.001
Reasoning errors per report, mean (SD)	0.4 (0.5)	0.4 (0.6)	0.88
Incompleteness (%)	63.53	44.31	<.001
Omission of information (%)	0.78	0.39	0.68
Hallucinations (%)	5.88	0.59	<.001
Misinformation (%)	7.84	1.96	<.001
Inconsistency (%)	0.59	0.39	>.99

^a^Chi-square tests were used for categorical measurements and Wilcoxon rank-sum tests for continuous measurements.

### Robustness

Using different prompt strategies introduces some variability in ChatGPT's responses to a given FFA report. Hence, we evaluated the robustness of ChatGPT with different language prompts through calculating the average diagnostic performance for 4 prompting methods: Direct, Step, Step (more detail), and Step (2-step) (Multimedia Appendix 1). The precision, recall, and *F*_1_-score, presented descriptively as mean (SD) values, were compared between Chinese and English prompts using Student *t* tests. As shown in Table 4, the results indicated significant differences in the recall (*P*=.03) and *F*_1_-score (*P*=.04) between Chinese and English prompts, while no significant difference was observed in the precision (*P*=.18). The mean (SD) *F*_1_-scores of ChatGPT with Chinese and English prompts were 70.02% (3.54%) and 76.47% (2.61%), respectively. In short, the diagnostic performance of ChatGPT with English prompts was better and more robust.

**Table 4:** The robustness of ChatGPT with various prompts in Chinese and English.

Diagnostic performance (%), mean (SD)	Chinese prompt	English prompt	*P* value
Precision	74.38 (1.49)	76.64 (2.10)	.18
Recall	68.78 (3.03)	78.71 (4.46)	.03
*F*_1_-score	70.02 (3.54)	76.47 (2.61)	.04

## Discussion

### Principal Findings

To the best of our knowledge, this is the first study to evaluate ChatGPT’s performance on non-English clinical text for retinal vascular disease diagnosis. We have developed a systematic evaluation scheme that encompasses objective diagnostic performance, professional inference abilities, and comparisons with the diagnostic ability of experts. Through large-scale experiments and analysis, we found the potential of LLMs as medical assistants to provide diagnosis, and identified challenges faced by ChatGPT in the field of health care, especially regarding language disparity.

Our results demonstrated that ChatGPT can preliminarily diagnose retinal vascular diseases based on Chinese FFA reports and achieved a high *F*_1_-score of 80.05% at best. However, the diagnostic performance of ChatGPT varied significantly among different diseases and prompting languages. The performance for common DR was significantly better than that for the more uncommon VKH, which is relatively low in incidence and more difficult to diagnose. Another noteworthy phenomenon is the language disparity of ChatGPT. Given that the development and validation of ChatGPT predominantly relied on English-centric data sets [31] and that non-English medical corpora are even more scarce, compared to with English prompts, ChatGPT exhibited a significant decline in diagnostic performance with Chinese prompts, with a 6.45% decrease in *F*_1_-score. This language disparity poses challenges for the effective application of ChatGPT in non-English clinical settings.

Meanwhile, the diagnosis accompanied by reasoning steps did not necessarily lead to performance improvement; *F*_1_-scores decreased by 6.59% for English prompts but increased by 5.14% for Chinese prompts. This disparity may be attributed to ChatGPT’s training being mainly on English corpora, with Direct-English prompts enabling a straightforward mapping from input to diagnosis. In contrast, Step-English prompts tended to bring more mistakes than benefits through multistep internal reasoning. However, for Chinese prompts, the scarcity of Chinese training data results in limited knowledge for disease diagnosis. Step-Chinese prompts, with the requirement of a reasoning process, can effectively compensate for incomplete and incorrect reasoning caused by limited knowledge, although they may introduce some noise. The performance gap between different diseases and prompting strategies demonstrates the potential unfairness brought by the overrepresentation of the major diseases, languages, and countries. This limitation hinders the global applicability of ChatGPT, particularly in non–English-speaking countries.

From the perspective of clinical practice, ChatGPT’s diagnostic performance still did not reach the level of ophthalmologists or even ophthalmology interns. It is worth noting that ChatGPT may be conservative in disease diagnosis. Despite the instruction restriction (must identify one), certain responses involved multiple conditions or indicated an inability to conclude based on existing information. Notably, although ChatGPT can derive a reasoning process to improve clinical interpretability, ophthalmologists identified some harmful mistakes, such as generating medical findings not mentioned in the original reports and quoting incorrect prior knowledge. More in-depth investigation and careful regulation are required before applying ChatGPT in the health care domain. Also, it is imperative to incorporate more extensive and higher-quality clinical data sets and knowledge into ChatGPT [32-34].

### Comparison to Prior Work

Prior work in using artificial intelligence (AI) for the automated diagnosis of retinal vascular disease has yielded promising outcomes [35]. However, since some hospitals struggle to produce qualified FFA reports [36] and require ophthalmologists with extensive clinical experience or retinal specialists, the majority of these studies have predominantly focused on analyzing FFA images. Ryu et al [37] introduced an end-to-end deep convolutional neural network–based method specifically designed for the automatic detection of DR and the assessment of lesion status. Similarly, Ding et al [38] proposed a pipeline for detecting retinal vessels in FFA images using deep neural networks. Moreover, Li et al [39] presented a weakly supervised learning-based method for detecting fluorescein leakage, eliminating the need for manual annotation of leakage areas. In contrast to research predominantly centered on lesion detection or specific disease diagnoses, Zhao et al [40] developed an AI system capable of automating image phase identification, diagnosing 4 different types of retinal diseases, and segmenting ischemic areas using FFA images. In our study, we used ChatGPT with 4 different prompting strategies based on FFA reports to diagnose a series of retinal diseases. Notably, when using an English prompt for direct diagnosis, ChatGPT exhibited impressive performance in the classification of retinal vascular diseases, requiring no additional training.

Beyond diagnostic accuracy, researchers have dedicated efforts to enhance the interpretability of disease diagnoses [41,42]. The widely used method for this purpose is heatmap visualization [38-40], used to accentuate characteristic regions crucial for disease diagnosis. This method may not capture the nuanced interplay of features critical for accurate diagnosis, leading to a potential loss of information and subtlety in the interpretative process. In this study, ChatGPT showed promise in enhancing the interpretability of disease diagnoses by explaining the process of diagnostic reasoning step by step. Its capacity to generate human-readable responses also allows for a more intuitive understanding of the AI diagnostic process.

ChatGPT has been used for various applications in clinical care and research. While numerous studies have demonstrated promising outcomes in complex medical tasks, including the United States Medical Licensing Exam (USMLE) [17,43], simplifying imaging reports for patients [27] and aiding decision-making [44,45], it is crucial to note that ChatGPT exhibits certain limitations. In the execution of the aforementioned tasks, ChatGPT occasionally produces errors, such as hallucinations or incomplete information [46]. However, the preceding studies were limited to the application and evaluation of ChatGPT solely within English medical contexts, neglecting an exploration of its effectiveness in non-English clinical scenarios. This study fills this gap by leveraging Chinese FFA reports to assess ChatGPT’s diagnostic performance and inference abilities for retinal vascular diseases in a non-English clinical environment and exploring the appropriate prompt languages and strategies.

### Limitations

Our study has several limitations. First, we did not fully utilize all the information available in clinical scenarios to conduct a diagnosis, such as more detailed FFA images, which may have reduced the diagnostic accuracy due to incomplete information. Since ChatGPT cannot analyze images, we will further evaluate the capabilities of multimodal models in subsequent research. Second, this study was not conducted in clinical practice. A prospective clinical trial can better examine an LLM’s clinical benefit; we leave this to our future work.

### Conclusions

This study conducted extensive experiments to evaluate the diagnostic capabilities of ChatGPT in retinal vascular diseases, including objective diagnostic performance and professional reasoning analysis evaluated by ophthalmologists. ChatGPT with English prompts for direct diagnosis performed best, achieving results close to the diagnostic performance of ophthalmology interns with 2 years of clinical experience. On the contrary, due to limited Chinese training data and knowledge, ChatGPT with Chinese prompts led to incomplete reasoning and poor diagnostic performance, which demonstrates that there is a significant language disparity in the application of ChatGPT in clinical environments. Additionally, although ChatGPT can derive a reasoning process with a low error rate, mistakes such as misinformation and hallucinations still exist, which will mislead the diagnose of retinal vascular diseases. This study generally reveals the potential of LLMs to serve as a helpful medical assistant to provide diagnosis in non-English clinical environments, but also demonstrates the potential limitations and the need to continually explore more robust LLMs in ophthalmology practice.

## Appendix

Multimedia Appendix 1 Example of the input and output of ChatGPT with various prompts.

## Abbreviations

AI: artificial intelligence
API: application programing interface
BRVO: branch retinal vein occlusion
CRVO: central retinal vein occlusion
DR: diabetic retinopathy
FFA: fundus fluorescein angiography
LLMs: large language models
SAHZU: The Second Affiliated Hospital of Zhejiang University
VKH: Vogt-Koyanagi-Harada disease

## Data Availability

Data will be made available for research purposes upon request. Data requests are to be directed to KJ.
