# Supplementary material for: Uncovering Language Disparity of ChatGPT on Retinal Vascular Disease Classification: Cross-Sectional Study
Source: J Med Internet Res. 2024 Jan 22;26:e51926. doi: 10.2196/51926 (PMC10845019; doi:10.2196/51926)
Supplement: Multimedia Appendix 1 [file jmir_v26i1e51926_app1.docx]

**Example of Input and Output of ChatGPT with Various Prompts**

| **Prompt strategy** | **Input** | **Output** | **Gold Standard** |
| --- | --- | --- | --- |
| Direct-Chinese | 您的任务是根据患者的眼底血管造影报告，确定患者的眼部状态。患者的眼部状态可能为：(A)正常，或是以下六种眼科疾病之一：(B)糖尿病视网膜病变, (C)年龄相关性黄斑变性, (D)中心性浆液性脉络膜视网膜病变, (E)视网膜分支静脉阻塞, (F)视网膜中央静脉阻塞, (G)Vogt-小柳原田综合症。请输出唯一正确的患者眼部状态选项。  患者的眼底血管造影术报告：左眼黄斑区可见斑驳状荧光，中间小片出血遮蔽荧光，周边可见点状高荧光，后期黄斑区可见荧光渗漏。 | 患者眼部状态为(C)年龄相关性黄斑变性。 | wetAMD/年龄相关性黄斑变性 |
| Direct-English | Your task is to determine which of the following eye conditions the patient has: (A) Normal, (B) Diabetic retinopathy, (C) Age-related macular degeneration, (D) Central serous chorioretinopathy, (E) Branch retinal vein occlusion, (F) Central retinal vein occlusion, (G) Vogt Koyanagi Harada Syndrome. Please output the only correct option of eye condition.  The patient's Fundus Fluorescein angiography report: 左眼黄斑区可见斑驳状荧光，中间小片出血遮蔽荧光，周边可见点状高荧光，后期黄斑区可见荧光渗漏。 | The patient has (C) Age-related macular degeneration. | wetAMD/年龄相关性黄斑变性 |
| Step- Chinese | 您的任务是分析患者的眼底血管造影报告，并确定患者的眼部状态是以下哪一种：(A)正常, (B)糖尿病视网膜病变, (C)年龄相关性黄斑变性, (D)中心性浆液性脉络膜视网膜病变, (E)视网膜分支静脉阻塞, (F)视网膜中央静脉阻塞, (G)Vogt-小柳原田综合症。最终诊断应该是给定的7个选项之一，不能同时涉及多项选择。请按以下格式提供输出：“我们逐步分析患者的眼底血管造影报告：{推理过程}，我们可以得出结论，患者的眼部状态为{眼部状态选项}”。  患者的眼底血管造影术报告：左眼网膜散在微血管瘤，毛细血管扩张渗漏，小片无灌注区，晚期荧光渗漏，黄斑区囊样积存。 | 我们逐步分析患者的眼底血管造影报告：报告显示左眼网膜散在微血管瘤、毛细血管扩张渗漏、小片无灌注区、晚期荧光渗漏、黄斑区囊样积存。这些症状表明患者可能患有糖尿病视网膜病变，因为微血管瘤和毛细血管扩张渗漏是糖尿病视网膜病变的典型表现，而无灌注区和荧光渗漏也是该病变的常见症状。黄斑区囊样积存则表明患者可能存在黄斑病变，这也是糖尿病视网膜病变的常见并发症之一。因此，我们可以得出结论，患者的眼部状态为糖尿病视网膜病变。 | DR/糖尿病视网膜病变 |
| Step- English | Your task is to analyze a patient's Fundus Fluorescein angiography report and determine exactly which of the following eye condition the patient is: (A) Normal, (B) Diabetic retinopathy, (C) Age-related macular degeneration, (D) Central serous chorioretinopathy, (E) Branch retinal vein occlusion, (F) Central retinal vein occlusion, (G) Vogt Koyanagi Harada Syndrome. The final diagnosis must be limited to one option from the given eight options and cannot involve multiple selections at once.Output should be structured as following format: "Based on the patient's Fundus Fluorescein angiography report, Let's think step by step: {reasoning process}. According to our comprehensive analysis, we can conclude that the patient's eye condition is {eye condition option}."  The patient's Fundus Fluorescein angiography report: 左眼网膜散在微血管瘤，毛细血管扩张渗漏，小片无灌注区，晚期荧光渗漏，黄斑区囊样积存。 | Based on the patient's Fundus Fluorescein angiography report, we can see that there are scattered microaneurysms in the left retina, as well as capillary dilation and leakage. There are also small areas of non-perfusion and late-stage fluorescence leakage, as well as cystic accumulation in the macular area. These findings are indicative of Diabetic retinopathy. Therefore, according to our comprehensive analysis, we can conclude that the patient's eye condition is Diabetic retinopathy. | DR/糖尿病视网膜病变 |
| Step- Chinese (more detail) | 作为一名经验丰富的临床眼科医生，你的任务是分析一名患者的眼底荧光血管造影报告，并确定他/她的眼睛状况。患者的眼睛可能是（A）正常, 或者患有以下其中一种眼病: (B)糖尿病视网膜病变, (C)年龄相关性黄斑变性, (D)中心性浆液性脉络膜视网膜病变, (E)视网膜分支静脉阻塞, (F)视网膜中央静脉阻塞, (G)Vogt-小柳原田综合症.  为了完成这项任务，你需要对报告中的每个描述都进行深入分析，利用医学知识和科学事实来指导推理过程。在分析过程中，请务必详细解释每一步推理的过程，让患者更容易了解您是如何得出结论的。虽然有时仅凭报告可能难以得出明确诊断，但你也应该选择最有可能的眼部状态而不是说无法诊断。最后，一定要找到尽可能多的可靠证据来支持你的推理过程。你的回答应该足够灵活，以便为你的推理过程提供各种相关的和创造性的解释。在您的回答中，请遵循以下结构："根据患者的眼底血管造影报告，让我们逐步思考: {推理过程}。根据我们的全面分析，我们可以得出结论，该患者的眼部状态是{眼部状态选项}。"  患者的眼底荧光血管造影报告: 左眼视盘旁出血遮蔽伴棉絮斑形成，后期血管渗漏。 | 根据患者的眼底血管造影报告，让我们逐步思考：  1. 左眼视盘旁出血遮蔽：这意味着在左眼视盘旁出现了出血，这可能是由于血管破裂或炎症引起的。这种情况可能与多种眼病有关。  2. 伴棉絮斑形成：棉絮斑是一种白色或灰色的斑块，通常与视网膜动脉或静脉阻塞有关。这种情况可能与视网膜分支静脉阻塞或视网膜中央静脉阻塞有关。  3. 后期血管渗漏：这意味着在后期，荧光素染料从血管渗漏到周围组织中。这种情况可能与糖尿病视网膜病变、年龄相关性黄斑变性、中心性浆液性脉络膜视网膜病变或Vogt-小柳原田综合症有关。  根据我们的全面分析，我们可以得出结论，该患者的眼部状态是视网膜分支静脉阻塞。这是因为左眼视盘旁出血和棉絮斑形成是视网膜分支静脉阻塞的典型表现。后期血管渗漏也支持了这一诊断。虽然其他眼病也可能导致类似的症状，但视网膜分支静脉阻塞是最有可能的诊断。 | BRVO/视网膜分支静脉阻塞 |
| Step- English (more detail) | As an experienced clinical ophthalmologist. Your task is to analyze a Chinese patient's Fundus Fluorescein angiography (FFA) report and determine his/her eye condition. The patient may have a normal eye (A), or one of the following eye diseases: (B) Diabetic retinopathy, (C) Age-related macular degeneration, (D) Central serous chorioretinopathy, (E) Branch retinal vein occlusion, (F) Central retinal vein occlusion, (G) Vogt Koyanagi Harada Syndrome.  To accomplish this task, you will need to conduct an in-depth analysis of each manifestation in the report, using medical knowledge and scientific facts to guide your reasoning process. As you progress through your analysis, be sure to explain each step of your reasoning process in detail, allowing patients to easily understand how you arrived at your conclusion. Although sometimes it may be difficult to reach an exact diagnosis from this report alone, you should also select the most likely eye condition rather than saying cannot diagnose. Finally, be sure to find as much reliable evidence as possible to support your reasoning process. Your answers should be flexible enough to provide various relevant and creative explanations for reasoning process.  In your response, please follow the structure below: 'Based on the patient's Fundus Fluorescein angiography report, Let's think step by step: {reasoning process}.  According to our comprehensive analysis, we can conclude that the patient's eye condition is {eye condition option}.'  The patient's Fundus Fluorescein angiography report: 左眼视盘旁出血遮蔽伴棉絮斑形成，后期血管渗漏。 | Based on the patient's Fundus Fluorescein angiography report, let's think step by step:  1. "左眼" indicates that the report is referring to the patient's left eye.  2. "视盘旁出血遮蔽" means that there is bleeding near the optic disc, which is the area where the optic nerve enters the eye.  3. "伴棉絮斑形成" suggests the presence of cotton wool spots, which are white patches on the retina caused by damage to nerve fibers.  4. "后期血管渗漏" indicates that there is leakage of blood vessels in the later stages of the angiography.  According to our comprehensive analysis, we can conclude that the patient's eye condition is most likely Diabetic Retinopathy (B). Diabetic Retinopathy is a complication of diabetes that affects the blood vessels in the retina, leading to bleeding, cotton wool spots, and leakage of blood vessels. The presence of bleeding near the optic disc and cotton wool spots in the patient's left eye, along with the later-stage leakage of blood vessels, are all consistent with the diagnosis of Diabetic Retinopathy. However, further examination and testing may be necessary to confirm the diagnosis. | BRVO/视网膜分支静脉阻塞 |
| Step- Chinese (2 steps) | 您的任务是根据患者的眼底血管造影报告和分析，确定患者的眼部状态是以下哪一种：(A)正常, (B)糖尿病视网膜病变, (C)年龄相关性黄斑变性, (D)中心性浆液性脉络膜视网膜病变, (E)视网膜分支静脉阻塞, (F)视网膜中央静脉阻塞, (G)Vogt-小柳原田综合症。请输出唯一正确的患者眼部状态选项。\n患者的眼底血管造影术报告：右眼视网膜中央静脉阻塞，视网膜静脉充盈迟缓，散在片状出血荧光遮蔽，小片无灌注区，毛细血管扩张渗漏。  分析：让我们逐步思考：1. 右眼视网膜中央静脉阻塞是报告中的主要发现，这意味着血液无法流动到视网膜的中央静脉，导致视网膜缺血和水肿。2. 视网膜静脉充盈迟缓是另一个重要的发现，这意味着血液在视网膜静脉中的流动速度减慢。3. 散在片状出血荧光遮蔽和小片无灌注区是视网膜缺血和水肿的表现，这些症状通常与视网膜病变有关。4. 毛细血管扩张渗漏也是视网膜病变的常见表现之一。  综上所述，根据患者的眼底血管造影报告，最终诊断应该是视网膜中央静脉阻塞。选项(F)视网膜中央静脉阻塞与报告中的发现完全吻合，因此是正确答案。  答案： | 视网膜中央静脉阻塞 | CRVO/视网膜中央静脉阻塞 |
| Step-English (2 steps) | According to a patient's Fundus Fluorescein angiography report and its analysis, your task is to determine exactly which of the following eye condition the patient is: (A) Normal, (B) Diabetic retinopathy, (C) Age-related macular degeneration, (D) Central serous chorioretinopathy, (E) Branch retinal vein occlusion, (F) Central retinal vein occlusion, (G) Vogt Koyanagi Harada Syndrome. The final diagnosis must be limited to one option from the given seven options and cannot involve multiple selections at once. Please output the only correct option of eye condition. The patient's Fundus Fluorescein angiography report: 右眼视网膜中央静脉阻塞，视网膜静脉充盈迟缓，散在片状出血荧光遮蔽，小片无灌注区，毛细血管扩张渗漏。  Analysis: Let's think step by step. The report states that the patient has "右眼视网膜中央静脉阻塞," which translates to "central retinal vein occlusion in the right eye." This rules out options A, B, C, D, E, and G, leaving us with option F, central retinal vein occlusion, as the final diagnosis.  Diagnosis: " | Central retinal vein occlusion | CRVO/视网膜中央静脉阻塞 |
